# Structural Damage Identification Based on Transmissibility in Time Domain

**DOI:** 10.3390/s22010393

**Published:** 2022-01-05

**Authors:** Yunfeng Zou, Xuandong Lu, Jinsong Yang, Tiantian Wang, Xuhui He

**Affiliations:** 1 School of Civil Engineering, Central South University, Changsha 410075, China; yunfengzou@csu.edu.cn (Y.Z.); 204811120@csu.edu.cn (X.L.); xuhuihe@csu.edu.cn (X.H.); 2 School of Traffic and Transportation Engineering, Central South University, Changsha 410075, China; wangtiantian@csu.edu.cn

**Keywords:** damage identification, transmissibility, time domain, finite element model updating, sensitivity

## Abstract

Structural damage identification technology is of great significance to improve the reliability and safety of civil structures and has attracted much attention in the study of structural health monitoring. In this paper, a novel structural damage identification method based on transmissibility in the time domain is proposed. The method takes the discrepancy of transmissibility of structure response in the time domain before and after damage as the basis of finite element model updating. The damage is located and quantified through iteration by minimizing the difference between the measurements at gauge locations and the reconstruction response extrapolated by the finite element model. Taking advantage of the response reconstruction method based on empirical mode decomposition, damage information can be obtained in the absence of prior knowledge on excitation. Moreover, this method directly collects time-domain data for identification without modal identification and frequent time–frequency conversion, which can greatly improve efficiency on the premise of ensuring accuracy. A numerical example is used to demonstrate the overall damage identification method, and the study of measurement noise shows that the method has strong robustness. Finally, the present work investigates the method through a simply supported overhanging beam. The experiments collect the vibration strain signals of the beam via resistance strain gauges. The comparison between identification results and theoretical values shows the effectiveness and accuracy of the method.

## 1. Introduction

Civil structures are faced with structural aging, adverse environmental impacts and other problems during operation, which will affect the safety and durability of the structure. Structural health monitoring has received attention [1,2,3]. Due to the complex form and large scale of civil structures, local damage is difficult to directly observe in daily operation processes. The initial damage may destroy the performance of the entire component; furthermore, some security incidents may occur. Therefore, regular inspection and condition assessment of an engineering structure are necessary so that early detection of any defect can be made and the safety and reliability of the structure can be determined.

Most existing damage identification methods rely on sensor systems [4,5]. The vibration information collected by the sensor is used to calculate the inverse problem to obtain the change in the structures to detect the damage. In terms of the algorithms used, damage identification methods can be classified into two types. One is data-driven methods, which typically fit data analysis models to the measured response data and then extract features sensitive to variations caused by damage and insensitive to operational and environmental variations, such as partial autoregressive models and their variants [6,7,8,9] and partial methods based on machine learning [10,11,12,13]. Although these methods do not require structural finite element (FE) models, it is difficult to accurately locate damage and provide limited information about damage severity based only on response data.

Another type of damage identification method is based on the structural model, which has been widely used due to its capacity to not only locate damage but also quantify the extent of damage. As a kind of model-based method, methods based on modal parameters [14,15,16,17,18,19,20,21,22,23] are very popular. Cui et al. [24] identified the strain modal parameters of structures under ambient excitation by combining the natural excitation technique based on the strain response with the eigensystem realization algorithm and then identified the damage through the detection index. Cancelli et al. [25] used stochastic subspace identification data to reconstruct the reduced-order stiffness matrix and locate and quantify the damage by a particle swarm optimization algorithm. Ghahremani et al. used blind source separation–sparse component analysis and frequency domain decomposition to calculate the modal information and detected the damage location and severity by solving a linear regression problem. However, the modal identification required by these methods relies on the accurate measurement response, and the complexity of the process of modal identification greatly affects the efficiency of damage identification. In this regard, damage identification based on FE model updating is a very effective method. Its core problem is to build a comparative data set to detect the damage. Some researchers have proposed methods combined with response reconstruction that avoid modal identification and have any analytic or numerical model of the structure. Zhang et al. [26] proposed a multilevel damage identification method, which used response reconstruction based on the Kalman filter to supplement the response data and improve the accuracy of damage identification. This method requires excitation information, which is very limited in practical applications. Pan and Yu [27] proposed a sparse-regularization-based method for detecting structural damage using structural responses caused by unknown moving forces. The measured responses are used as inputs to estimate the reconstructed responses with the help of the transmissibility matrix, which is applied to establish the minimization problem. However, these methods are carried out in the frequency domain, which requires considerable time–frequency conversion in the identification process and increases the calculation time and cost. Thus, this study aims to propose a damage identification method based on response reconstruction with more efficiency.

In contrast with frequency-domain reconstruction methods, time-domain methods directly solve the modal characteristics and establish transmissibility. The structural response reconstruction method based on empirical mode decomposition (EMD) in the time domain [28,29,30] has proven to be a very efficient method in terms of computational cost and is very suitable for various dynamic response reconstructions based on the different types of sensor measurements. This method does not need to take time–frequency conversion for the response signal. To the best of the authors’ knowledge, the transmissibility of response in the time domain has not been applied to damage identification in the literature before. If the structural parameters are changed due to structural damage, the response calculated by the original transmissibility is bound to be different from the real response, which can be used to detect the structural damage combined with FE model updating [31,32].

In this paper, combined with response reconstruction based on EMD, a kind of damage identification method based on transmissibility in the time domain is proposed. The method takes the discrepancy of transmissibility of the structure response in the time domain before and after damage as the basis of FE model updating, and then the damage location and damage degree are obtained through iteration. The solution process of the discrepancy vector and sensitivity matrix for FE model updating is given in this paper. The method does not need to obtain excitation information, which reduces the cost and improves the applicability in civil structures. In addition, the identification is carried out in the time domain without modal identification and frequent time–frequency conversion, which can greatly improve efficiency on the premise of ensuring accuracy. The overall method is demonstrated through a numerical example, and the effect of measurement noise is further studied. Then, the proposed method is experimentally investigated on a simply supported overhanging beam. The beam is excited with an impulse hammer, and its vibration signals are captured by resistance strain gauges bonded to the upper surface of the beam.

The paper is organized as follows: In Section 2, the basic principle of transmissibility of structural response in the time domain is briefly introduced. In Section 3, the detailed solution process of FE model updating based on transmissibility in the time domain is presented. In Section 4, through the multiple damage scenarios of a simply supported overhanging beam model, the correctness and effectiveness of the proposed method are verified, and the influence of different levels of measurement noise on the identification results is studied. In Section 5, the experimental beam corresponding to the simulation is used to prove the feasibility of the method. The conclusions are discussed in Section 6.

## 2. Transmissibility of Strain in Time Domain Based on Empirical Mode Decomposition

The difference between the measured dynamic response and the reconstruction response extrapolated by the FE model can be subject to structural damage identification due to the change in transmissibility before and after damage. The response in the domain measured by the sensors can be decomposed by using the method of empirical mode decomposition (EMD) with intermittent criteria [28,29]. The strain response vector can be expressed as:

(1) $$\varepsilon =\Psi q=\sum_{i=1}^{p}\eta_{i}$$

where **Ψ** is the strain modal matrix; **q** is the modal coordinate vector; $\eta_{i}$ is the *i*-th single-frequency modal response of **ε**; and *p* is the selected mode. **Ψ** can be obtained by transformation of the mode shape matrix **Φ**. The mode shape of the structure can be obtained by solving the eigenvalue problem:

(2) (K−ΛM)Φ=0

where **K** and **M** are global stiffness (mass) matrices of the structure; **Λ** is a diagonal matrix, whose diagonal elements are all the square values of the natural vibration frequencies of the structure. Under the premise of small deformation, the strain mode in the element can be calculated as follows:

(3) $$\Psi^{\left(l\right)}=B^{\left(l\right)}\cdot \Phi^{\left(l\right)}$$

in which $B^{\left(l\right)}$ is the strain-displacement matrix of the *l*-th element, which can be calculated by the differential operator and the shape function matrix of the *l*-th element. $\Phi^{\left(l\right)}$ contains the displacement mode shape index of the *l*-th element. Based on the modal analysis, the relationship between the modal responses of the two locations in the structure is as follows:

(4) $$\psi_{aj}/\psi_{bj}=\eta_{aj}/\eta_{bj}$$

in which subscripts *a* and *b* denote the degree of freedom (DOF) index of the strain mode and subscript *j* denotes the mode. All modal responses are set as matrices with dimensions (1 × *Nt*), and *Nt* denotes the number of time points of the collected response. The modal response at location b can be expressed by the modal response at location *a* as:

(5) $$\eta_{(a,b)j}^{r}=\eta_{aj}^{m}\frac{\psi_{aj}}{\psi_{bj}}$$

where $\psi_{aj}/\psi_{bj}$ is called the transmissibility of the response from location *a* to location *b*. The subscript (*a*,*b*)*j* means that the *j*-th modal response at location *a* is used to reconstruct the *j*-th modal response at location *b*. The superscripts *r* and *m* denote the reconstructed and measured values, respectively. It should be noted that the calculation of the response here only operates on the same mode response.

## 3. Finite Element Model Updating Based on Modal Response Transmissibility

### 3.1. Establishment of Objective Function

The rationale underlying FE model updating for damage identification is to seek the relevant parameters corresponding to the damage state by minimizing the difference between the measured data of the actual structure and the analysis data of the FE model during the optimization process so that the damage can be located and quantified. Considering the change of structural characteristics caused by damage, the objective function of damage identification is regarded as the problem of minimizing the discrepancy of reconstruction responses before and after structural damage:

(6) $$J(\alpha )={\|W(\eta_{d}^{r}-\eta_{f}^{r}(\alpha ))\|}_{2}^{2}$$

where $\eta_{d}^{r},\eta_{f}^{r}(\alpha )\in {ℜ}^{Nr\cdot Nt\times 1}$ are the modal response vectors derived from the transmissibility of the damaged structure and the FE model, respectively; $\alpha \in {ℜ}^{n}$ is the damage factor vector; *Nr* and *n* are the number of transmissibilities and the number of damage parameters involved in damage identification, respectively; **W** is a diagonal weighting matrix whose diagonal value can be set as the reciprocal of the variance of the structural modal response; and ‖⋅‖ denotes the Frobenius norm. Modal response data of the corresponding mode of two locations are used in each calculation. Equation (6) is the nonlinear function of damage factor **α**, and the gradient descent optimization method is usually used to solve the minimization, such as the Gauss–Newton iteration method:

(7) $$S_{\alpha }^{k}\Delta \alpha^{k+1}=\Delta \eta^{r,k}$$

in which $S_{\alpha }^{k}=\partial \eta^{r,k}(\alpha^{k})/\partial \alpha^{k}$ is the sensitivity matrix of the derived value to the damage factor in the *k*-th iteration; $\Delta \alpha^{k+1}$ is the damage factor increment obtained in the *k*-th iteration; and $\Delta \eta^{r,k}=\eta_{d}^{r}-\eta_{f}^{r,k}(\alpha^{k})$ is the discrepancy vector of the modal response calculated from the transmissibility of the damaged structure and the undamaged FE model in the initial state. $\alpha^{k}=\sum_{i=1}^{k}\Delta \alpha^{i}$ is the cumulative damage factor.

### 3.2. Establishment of Discrepancy Vectors

The discrepancy vector $\Delta \eta^{r,k}$ in Equation (7) can be assembled according to rows as:

(8) $$\begin{array}{l} \Delta \eta^{r,k}=[(\Delta \eta_{1}^{r,k}{)}^{T}\cdots {\left(\Delta \eta_{i}^{r,k}\right)}^{T}\cdots {\left(\Delta \eta_{Nr}^{r,k}\right)}^{T}{]}^{T}\\ \Delta \eta_{i}^{r,k}=[\Delta \eta_{i}^{r,k}(t_{1})\;\Delta \eta_{i}^{r,k}(t_{2})\cdots \Delta \eta_{i}^{r,k}(t_{Nt}){]}^{T} \end{array}$$

A certain component $\Delta \eta_{i}^{r,k}$ of the discrepancy vector can be calculated by the following equation:

(9) $$\Delta \eta_{i}^{r,k}=\eta_{d,i}^{r}-\eta_{f,i}^{r,k}(\alpha^{k})$$

where subscript *f* of $\eta_{f,i}^{r,k}(\alpha^{k})$ denotes the modal response calculated from the strain mode of the FE model; subscript i=1,2,…,Nr denotes the modal response component corresponding to the *i*-th transmissibility in the difference vector; and subscript d of $\eta_{d,i}^{r}$ denotes the modal response derived from the strain mode of the damaged structure. In general, the strain mode of the damaged structure is unknown. To calculate $\Delta \eta_{i}^{r,k}$, the component $\Delta \eta_{(a,b)j}^{r,k}$ in $\Delta \eta_{}^{r,k}$ is taken as an example. The measured value $\eta_{bj}^{m}$ of the modal response at location *b* is used to approximately substitute for the calculated value of the modal response in damage. $\Delta \eta_{(a,b)j}^{r,k}$ can be expressed as:

(10) $$\Delta \eta_{(a,b)j}^{r,k}=\eta_{d,(a,b)j}^{r}-\eta_{f,(a,b)j}^{r,k}(\alpha^{k})=\eta_{bj}^{m}-\eta_{aj}^{m}\frac{\psi_{bj}^{f}}{\psi_{aj}^{f}}$$

The superscript *f* of $\psi_{aj}^{f}$ and $\psi_{bj}^{f}$ denotes that the strain mode is obtained by solving the FE model. FE model updating seeks the optimal solution by minimizing the difference between the FE model and the damage model. To quantify model differences to control cycles, the relative error (RE) that reflects the extent to which the reconstructed response deviates from the measured response is defined as

(11) $$RE=\frac{\sum_{i=1}^{Nr\times Nt}{\left(\eta_{f,(a,b)j}^{r,k}(i)-\eta_{bj}^{m}(i)\right)}^{2}}{\sum_{t=1}^{n}{\left(\eta_{bj}^{m}(i)\right)}^{2}}$$

Convergence is considered to be achieved when the criterion RE≤tol is met, where *tol* denotes the tolerance value that is set equal to 1.0 × 10^−6^ in this study.

### 3.3. Establishment of Sensitivity Matrix

In Equation (7), $S_{\alpha }^{k}$ is obtained by row assembly of the sensitivity matrices corresponding to each transmissibility:

(12) $$S_{\alpha }^{k}={\left[{\left(S_{\alpha ,1}^{k}\right)}^{T}\cdots {\left(S_{\alpha ,i}^{k}\right)}^{T}\cdots {\left(S_{\alpha ,Nr}^{k}\right)}^{T}\right]}^{T}$$

The component $S_{\alpha ,(a,b)j}^{k}$ corresponding to component $\Delta \eta_{(a,b)j}^{r,k}$ in $\Delta \eta_{}^{r,k}$ can be specifically expressed as:

(13) $$S_{\alpha ,(a,b)j}^{k}=\partial \eta_{(a,b)j}^{r,k}(\alpha^{k})/\partial \alpha^{k}=\left[\frac{\partial \eta_{(a,b)j}^{r,k}(\alpha_{1}^{k})}{\partial \alpha_{1}^{k}}\cdots \frac{\partial \eta_{(a,b)j}^{r,k}(\alpha_{l}^{k})}{\partial \alpha_{l}^{k}}\cdots \frac{\partial \eta_{(a,b)j}^{r,k}(\alpha_{n}^{k})}{\partial \alpha_{n}^{k}}\right]$$

where *l =* 1, 2, …, *n* denotes the damage factor order involved in identification. According to Equation (9), the component $\partial \eta_{(a,b)j}^{r,k}(\alpha_{l}^{k})/\partial \alpha_{l}^{k}$ in Equation (13) can be calculated as follows:

(14) $$\frac{\partial \eta_{(a,b)j}^{r,k}(\alpha_{l}^{k})}{\partial \alpha_{l}^{k}}=\eta_{aj}^{m}\frac{\psi_{bj}^{f}\frac{\partial \psi_{aj}^{f}}{\partial \alpha_{l}^{k}}-\psi_{aj}^{f}\frac{\partial \psi_{bj}^{f}}{\partial \alpha_{l}^{k}}}{{\left(\psi_{aj}^{f}\right)}^{2}}=\eta_{aj}^{m}\frac{\psi_{bj}^{f}\frac{\partial (B_{a}^{\left(ka\right)}\Phi_{}^{f,(ka)})}{\partial \alpha_{l}^{k}}-\psi_{aj}^{f}\frac{\partial (B_{b}^{\left(kb\right)}\Phi_{}^{f,(kb)})}{\partial \alpha_{l}^{k}}}{{\left(\psi_{aj}^{f}\right)}^{2}}$$

where *ka* and *kb* denote the element order to which the measured locations *a* and *b* belong, respectively (locations *a* and *b* can belong to the same element). $B_{}^{\left(ka\right)}$ and $B_{}^{\left(kb\right)}$ are the strain-displacement matrices corresponding to the elements to which the measured locations *a* and b belong. For a certain location in the structure, the strain-displacement matrix is a constant matrix. $\Phi_{}^{f,(ka)}$ and $\Phi_{}^{f,(kb)}$ are the *j*-th mode shape vectors contained in the elements to which locations *a* and *b* belong, which are solved by the FE model method.

It is necessary to solve the derivative of the mode shape corresponding to the damage factor at first. The derivative of the *j*-th mode shape index $\varphi_{ij}$ corresponding to the *i*-DOF of the structure corresponding to the damage factor can be solved by the iterative method [33,34]:

(15) $$\frac{\partial \varphi_{ij}}{\partial \alpha_{l}^{k}}=\sum_{s=1}^{q}g_{sj}\varphi_{is}$$

in which *q* means that the first *q* mode is selected to iteratively solve the mode shape sensitivity. In Equation (15), the solution is as follows:

(16) $$g_{sj}=\{\begin{array}{c} \frac{1}{\lambda_{j}-\lambda_{s}}\varphi_{s}^{T}(\frac{\partial K}{\partial \alpha_{l}^{k}}-\frac{\partial \lambda_{s}}{\partial \alpha_{l}^{k}}M-\lambda_{s}\frac{\partial M}{\partial \alpha_{l}^{k}})\varphi_{j},\;(j\neq s)\\ -\frac{1}{2}\varphi_{j}^{T}\frac{\partial M}{\partial \alpha_{l}^{k}}\varphi_{j},\;(j=s) \end{array}$$

where $\lambda_{j}$ and $\lambda_{s}$ are the *j*(*s*)-th eigenvalues of the structure, which are numerically equal to the square of the natural vibration frequency of the structure. $\varphi_{j}$ and $\varphi_{r}$ represent the *j*(s)-th eigenvectors obtained by solving the characteristic problem, that is, the *j*-th mode shapes of the structure. The derivative of $\lambda_{s}$ corresponding to the damage factor is solved as follows:

(17) $$\frac{\partial \lambda_{s}}{\partial \alpha_{l}^{k}}=\varphi_{s}^{T}(\frac{\partial K}{\partial \alpha_{l}^{k}}-\lambda_{s}\frac{\partial M}{\partial \alpha_{l}^{k}})\varphi_{s}$$

There is a linear relationship between the strain mode and element mode shape. Thus, the sensitivity matrix for each iteration can be calculated by Equation (14).

The proposed damage identification method based on the FE model obtains accurate results by comparing the measured signal with the reconstructed signal. Therefore, the fast and accurate acquisition of reconstructed signals is the main source affecting the calculation accuracy and efficiency. In this paper, a damage identification method based on more efficient response reconstruction is proposed, which can greatly improve the efficiency on the premise of ensuring accuracy.

## 4. Numerical Example

### 4.1. The Model of Simply Supported Overhanging Beam

**Figure 1:**
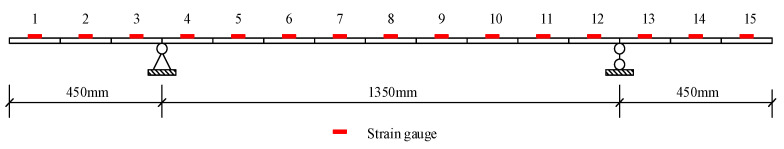
The model of simply supported overhanging beam.

The simulation case of a simply supported overhanging beam (2.25 m × 3 cm × 1 cm) is studied to illustrate the effectiveness of the proposed method. The numerical model for identification is established in MATLAB 2020b, which consists of 16 nodes and 15 0.15 cm length elements. It is assumed that the strain gauges measuring strain responses are set at the top center of each element, as shown in Figure 1. The damping effect is simulated by 1% modal damping. The integral time step is 1/1000 s, and the duration is 30 s. The strain response was collected at the top surface in the middle of each element. The dynamic response recorded by the sensor is calculated using a first-order hold (FOH) state space.

### 4.2. Damage Scenario Simulation

In this case, structural damage is achieved by reducing the element stiffness and mass. It is assumed that the stiffness and mass matrix are linearly dependent on the damage factor α and can be expressed as:

(18) $$\begin{array}{l} K(\alpha )=K_{u}+\sum_{l=1}^{n}\alpha_{l}^{k}K_{l},\;(-1\leq \alpha_{l}^{k}\leq 0)\\ M(\alpha )=M_{u}+\sum_{l=1}^{n}\alpha_{l}^{m}M_{l},\;(-1\leq \alpha_{l}^{m}\leq 0) \end{array}$$

where $K_{u}$ and $M_{u}$ are the global stiffness (mass) matrix of the undamaged structure; $K_{l}$ and $M_{l}$ are the contributions of the *l-*th element to the global stiffness (mass) matrix; and $\alpha_{l}^{k}$ and $\alpha_{l}^{m}$ are the equivalent stiffness (mass) damage factors of the *l*-th element. The equivalent damage equates the local section loss, material degradation and other factors to the stiffness (mass) reduction of the whole element, making the dynamic response characteristics of the FE model as close as possible to the damaged structure.

**Figure 2:**
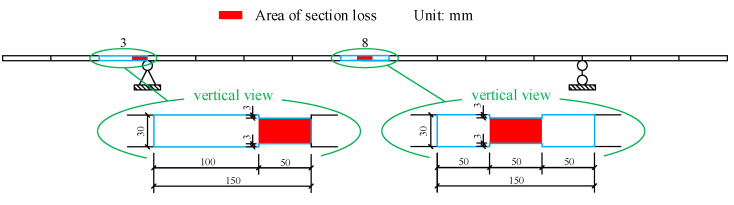
The schematic diagram of damages.

In this study, the damage is simulated by uniformly reducing the width of the rectangular section in a certain length of the beam segment in Element 3 and Element 8, as shown in Figure 2. Damage scenarios are shown in Table 1. The specific value of equivalent damage cannot be determined directly in the beam model. The damage identification method based on strain mode [24,35] is used to reverse calculate the damage. The stain mode of 15 elements before and after damage is used for damage identification. To obtain accurate equivalent damage, only the preset damaged elements are identified, not the other elements. The calculation results of equivalent damage are shown in Table 2. The results serve as a reference for the damage identification described below.

### 4.3. Damage Identification Based on Transmissibility in Time Domain

**Figure 3:**
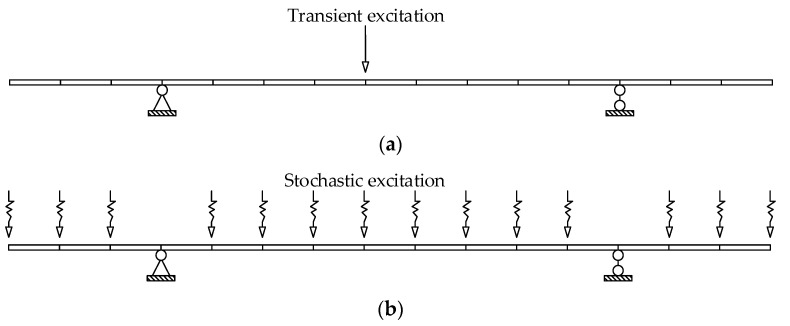
Schematic diagram of excitation applied on the beam: (**a**) transient excitation; (**b**) stochastic excitation.

**Figure 4:**
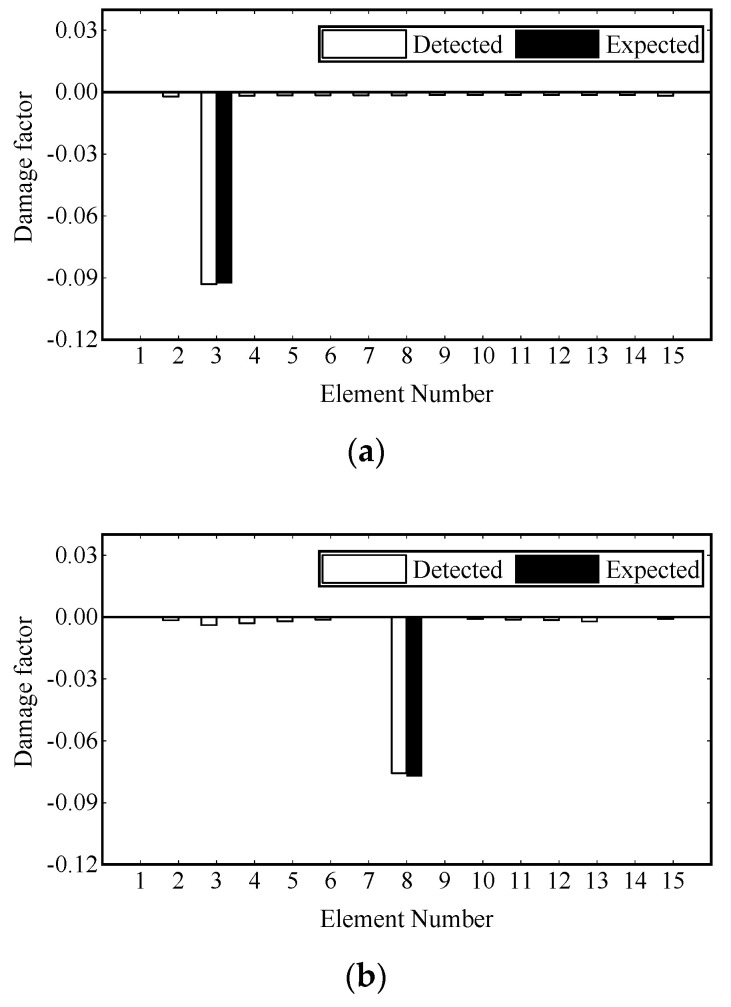

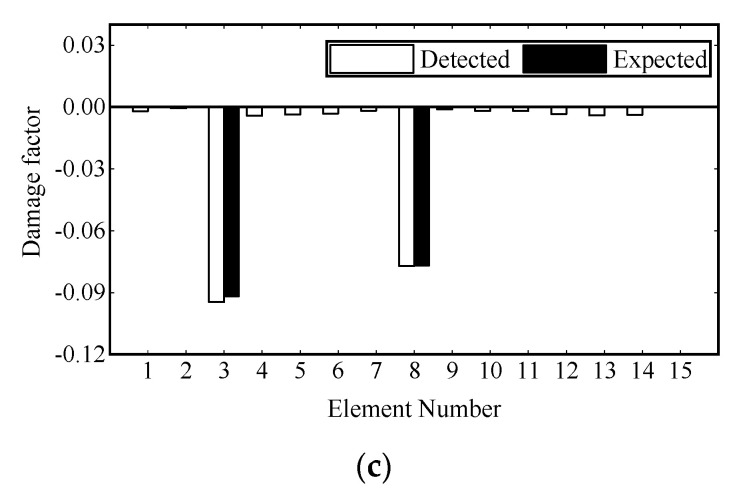
Damage identification results under transient excitation: (**a**) scenario 1; (**b**) scenario 2; (**c**) scenario 3.

**Figure 5:**
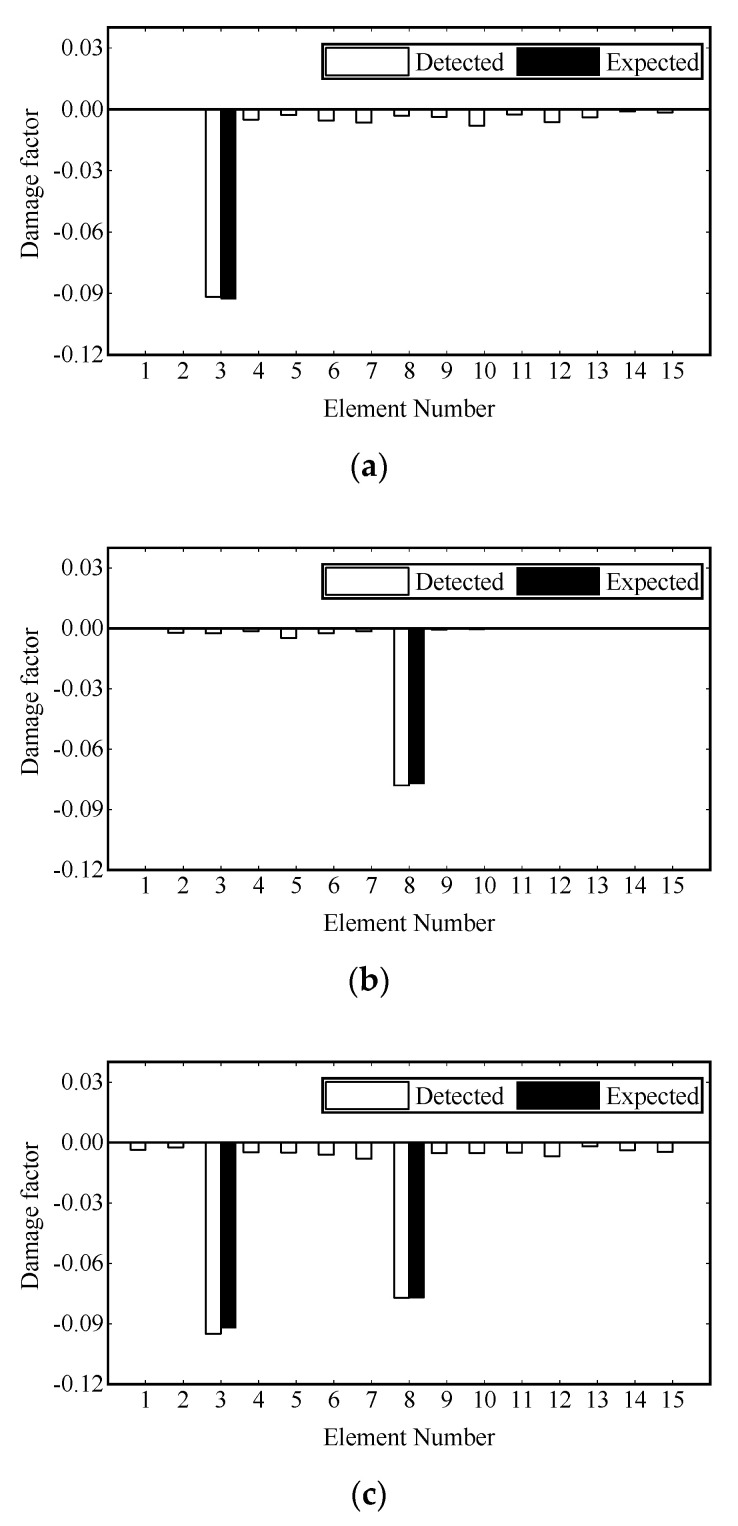
Damage identification results under stochastic excitation: (**a**) scenario 1; (**b**) scenario 2; (**c**) scenario 3.

Damage identification is carried out under transient excitation and stochastic excitation, respectively. The transient excitation is applied to the vertical DOF of Node 8, as shown in Figure 3a. The stochastic excitation is simulated by the white noise filtered by a sixth-order low-pass Butterworth filter and applied to the vertical degrees of freedom of multiple nodes, as shown in Figure 3b. The first modal responses are extracted from the strain data, and one second process is truncated for damage identification. Then the damage is identified by the adaptive Tikhonov regularization method [36]. The identification results are shown in Figure 4 and Figure 5, where all damages are successfully identified, and the detected damage factors of undamaged elements are close to zero.

### 4.4. Influence of Noise on Damage Identification

**Figure 6:**
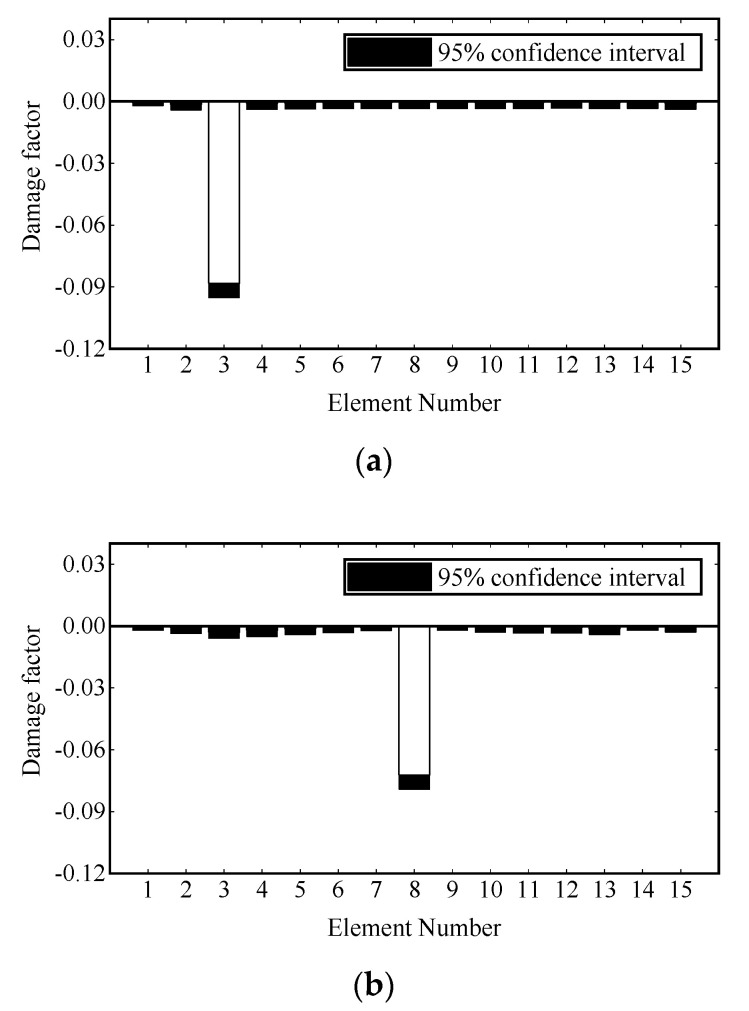

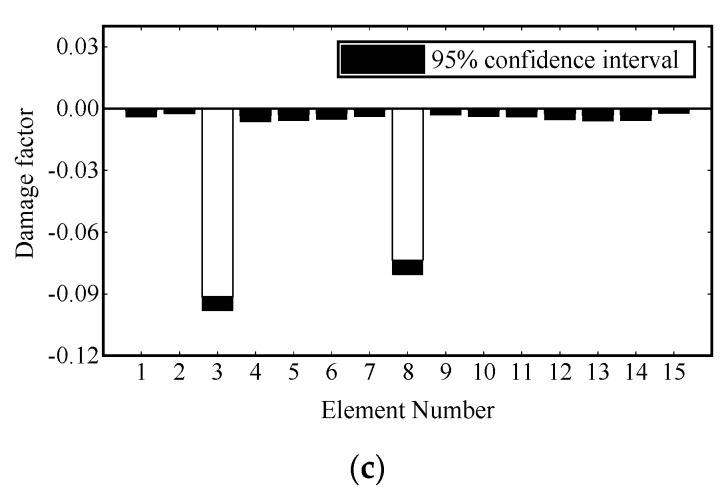
Damage identification results under 5% noise: (**a**) scenario 1; (**b**) scenario 2; (**c**) scenario 3.

**Figure 7:**
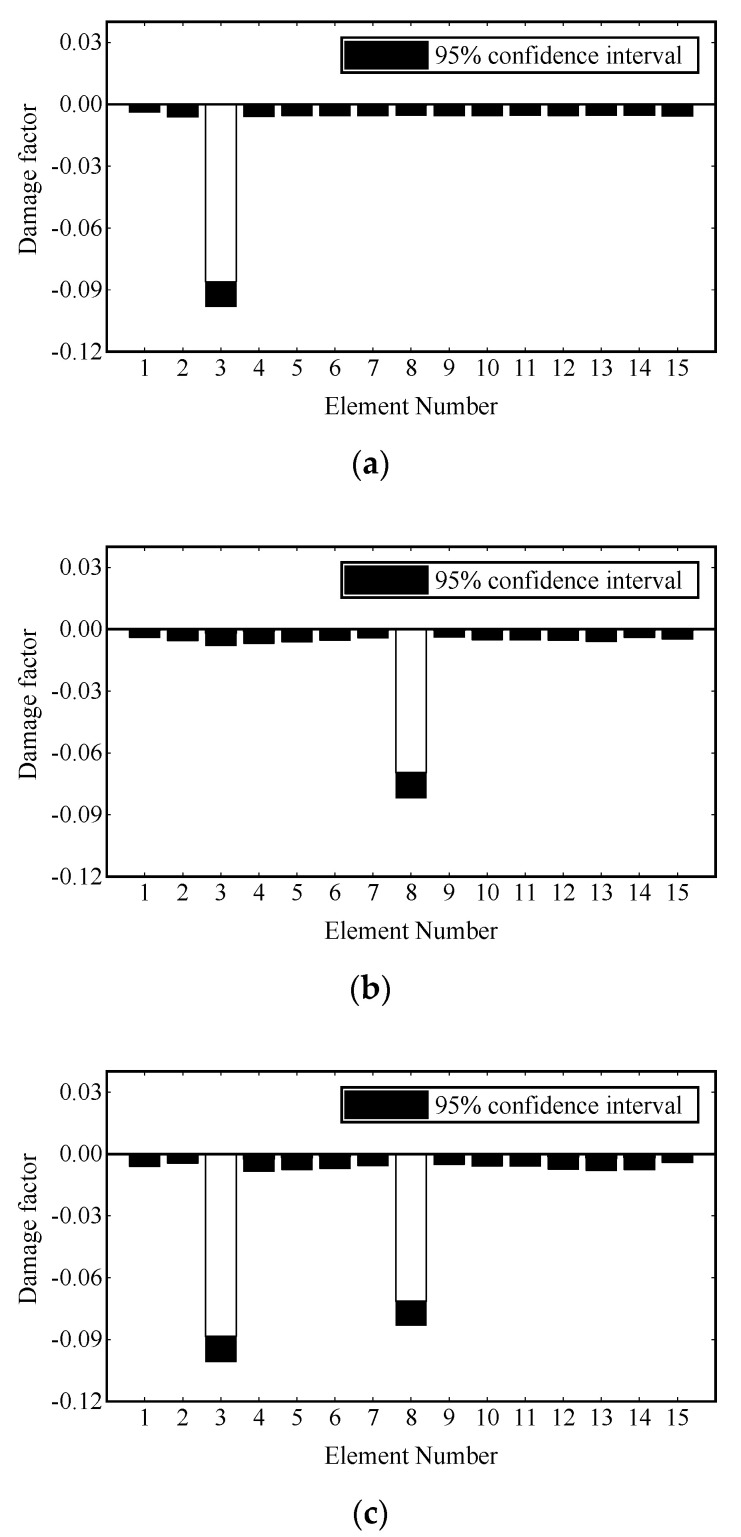
Damage identification results under 10% noise: (**a**) scenario 1; (**b**) scenario 2; (**c**) scenario 3.

**Figure 8:**
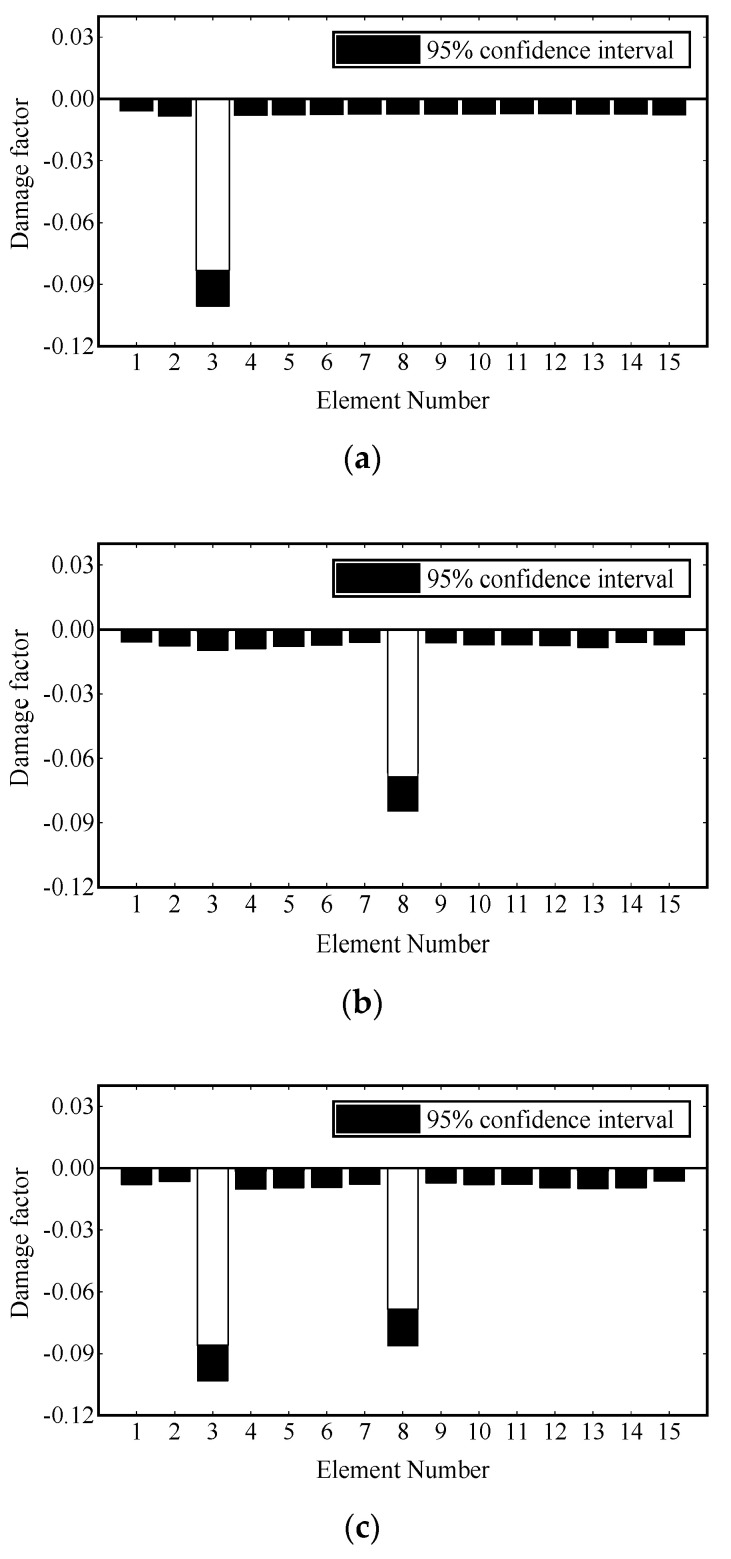
Damage identification results under 20% noise: (**a**) scenario 1; (**b**) scenario 2; (**c**) scenario 3.

Noise inevitably exists in the measurement of structural response signals. In this study, a normal random process with zero mean value and unit standard deviation was added to the strain signal under transient excitation as:

(19) $$\varepsilon_{noi}=\varepsilon_{cal}+E_{p}\mathrm{var}(\varepsilon_{cal})N_{oise}$$

where $\varepsilon_{noi}$ is the strain response vector after adding noise; $\varepsilon_{cal}$ is the calculated strain response vector; $E_{p}$ is the noise level; var(⋅) denotes the standard deviation of the time history of vector $\varepsilon_{cal}$; and $N_{oise}$ is a normal random process with zero mean and unit standard deviation. Considering the randomness of noise, when studying the influence of noise on damage identification, 1000 control groups are set for all damage scenarios. Measurement noise levels of 5%, 10% and 15% are added to the strain of each control group. Because the adaptive Tikhonov regularization limits the identified results to less than 0, it is considered that the identification values of all damage factors in the control groups conform to the gamma distribution. Finally, the 95% confidence intervals of the identification results under different noise levels and damage scenarios are calculated, which are shown in Figure 6, Figure 7 and Figure 8.

In Figure 6, 5% noise causes a fluctuation of approximately 0.007 for the damage factor identification of the preset damaged element, while the fluctuation range of other elements is less than 0.003. In Figure 7, 10% noise causes a fluctuation of approximately 0.012 for the damage factor identification of the preset damaged element, while the fluctuation range of the remaining elements is less than 0.007. In Figure 8, 15% noise causes the damage factor identification of the preset damaged element to fluctuate by approximately 0.018, while the fluctuation range of the remaining elements is less than 0.005. Overall, noise will affect the performance of the proposed damage identification method to some extent, but even if the noise level reaches 15%, the identification error is small, and the damaged elements can still be clearly located. However, the measurement noise in actual engineering is usually less than 10%. The proposed method has strong robustness to noise.

## 5. Experimental Investigation

### 5.1. Experimental Setup

**Figure 9:**
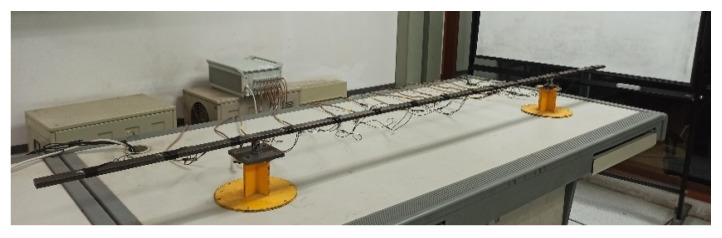
Laboratory test setup.

The same simply supported overhanging beams as in the simulation case are studied in the laboratory. There are four experimental beams in total including three damaged beams corresponding to three damage scenarios in the simulation case and one intact beam for comparison. All beams are made of Q235 steel. The setup in the laboratory is shown in Figure 9.

**Figure 10:**
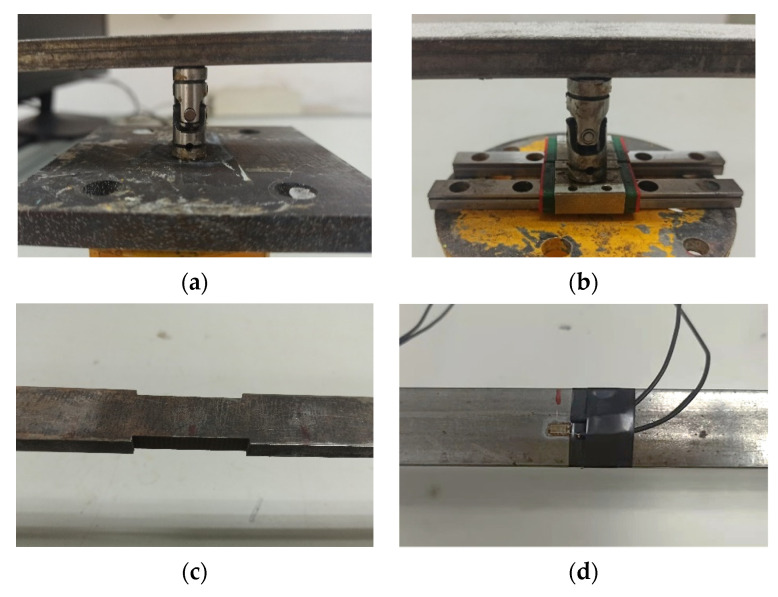

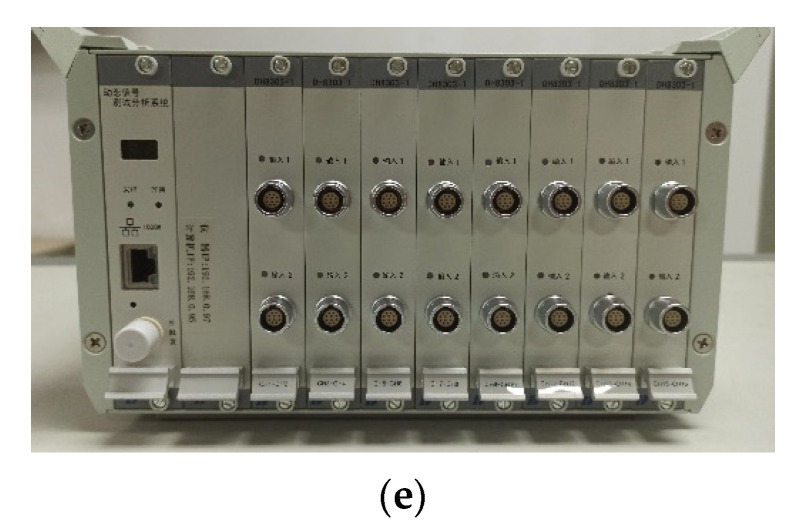
Experimental instruments: (**a**) fixed hinge support; (**b**) sliding hinge support; (**c**) rectangular notches; (**d**) strain gauge; (**e**) DH8303 dynamic signal test and analysis system.

The beam is mounted on a fixed hinge support and a sliding hinge support, as shown in Figure 10a,b, respectively. The fixed hinge support is simulated by two universal joints and fixed on the steel base. The steel base is cemented and fixed with the test bench to ensure that only the rotation freedom of the beam around the transverse axis is free at its location. Based on that, a slide rail is added between the universal joint and the steel base to simulate the sliding hinge support, which releases the longitudinal DOF of its location. Damage is generated by cutting equally at both sides of the beam on the element region (Figure 10c), and their damage characteristics are the same as those in the simulation case. The beam is excited with an impulse hammer at Node 6 to simulate the impulse force.

Resistance strain gauges are employed in this study to obtain strain measurements, as shown in Figure 10d. They are pasted at the top center of each element. The resistance strain gauges are of the same type, whose specifications are listed in Table 3. The Wheatstone 1/4 bridge is used to connect each strain gauge to convert the strain responses into an electrical signal through resistance change. DH8303 dynamic signal test and analysis system is applied to collect signals, as shown in Figure 10e.

### 5.2. Accuracy Detection of FE Model

The similarity of vibration characteristics between the FE model and experimental beam has a significant impact on damage identification. In the experimental beam mentioned in the previous section, the strain gauge and connecting line are light, and their additional stiffness and additional mass are ignored.

**Figure 11:**
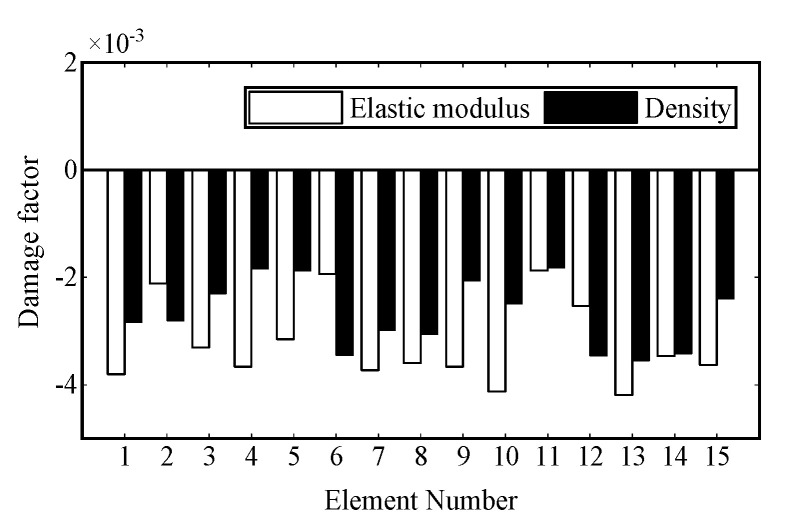
Identified results in the intact model.

For elastic modulus *E* and density *ρ*, if they are regarded as uniformly distributed along the whole beam, they are linear with the stiffness matrix and mass matrix, respectively. In this case, the change in *E* and *ρ* is equivalent to the stiffness matrix and mass matrix being multiplied by a single value, respectively, so that the strain modes calculated by Equations (2) and (3) remain unchanged, and the damage identification results will not be affected. Therefore, *E* and *ρ* are used as local variables to test whether they are evenly distributed or whether other error factors exist. The elastic modulus factor $\alpha_{l}^{E}=(E_{l}^{r}-E_{l}^{f})/E_{l}^{f}$ and the density factor $\alpha_{l}^{\rho }=(\rho_{l}^{r}-\rho_{l}^{f})/\rho_{l}^{f}$ are set, where the subscription *l* denotes the *l*-th element and the superscription *r* and *f* denote the real value and the value of the FE model, respectively. Strain gauges are placed along the whole beam on the intact beam, and the parameters are obtained according to the damage identification method proposed in this paper. The results in Figure 11 show that the identified values of the elastic modulus factor and density factor of each element are very small, and thus it can be considered that they are evenly distributed along the whole beam, and damage identification can be carried out directly without model updating.

### 5.3. Damage Identification

**Figure 12:**
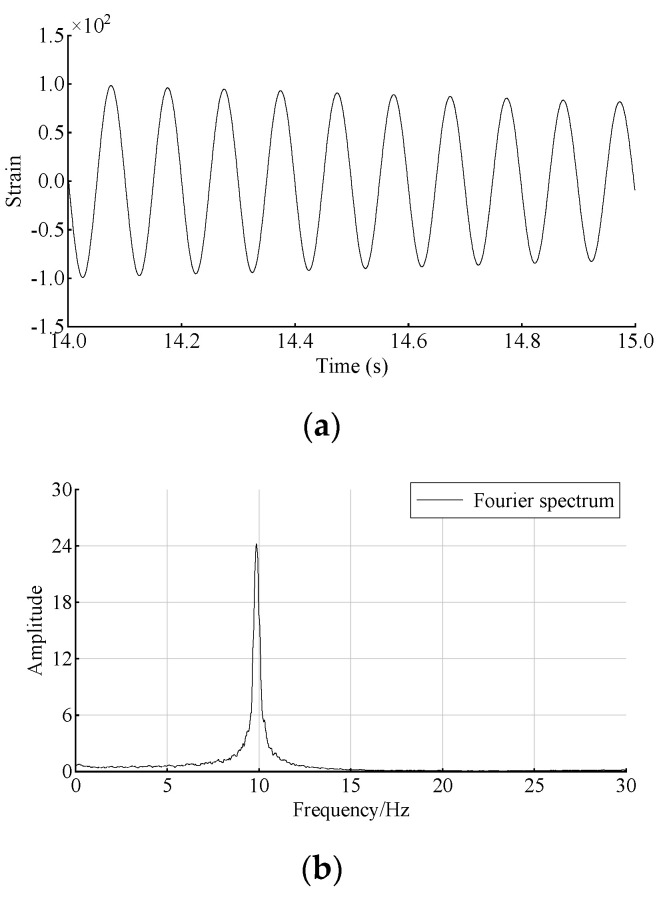
Time- and frequency-domain responses at Node 23 in damage scenario 2: (**a**) response in time domain; (**b**) response in frequency domain.

Measured responses from strain gauges on hammer excitation of the beam are used in the damage identification. Figure 12a,b show the dynamic response at Node 23 in damage scenario 2 in the time domain and frequency domain, respectively. The sampling frequency is set to 1 kHz, and the data of 1 s during free vibration are extracted for damage identification. Accordingly, the passband frequency of the bandpass filter is set to 8 Hz and 11 Hz, and the stopband frequency is set to 6 Hz and 13 Hz.

**Figure 13:**
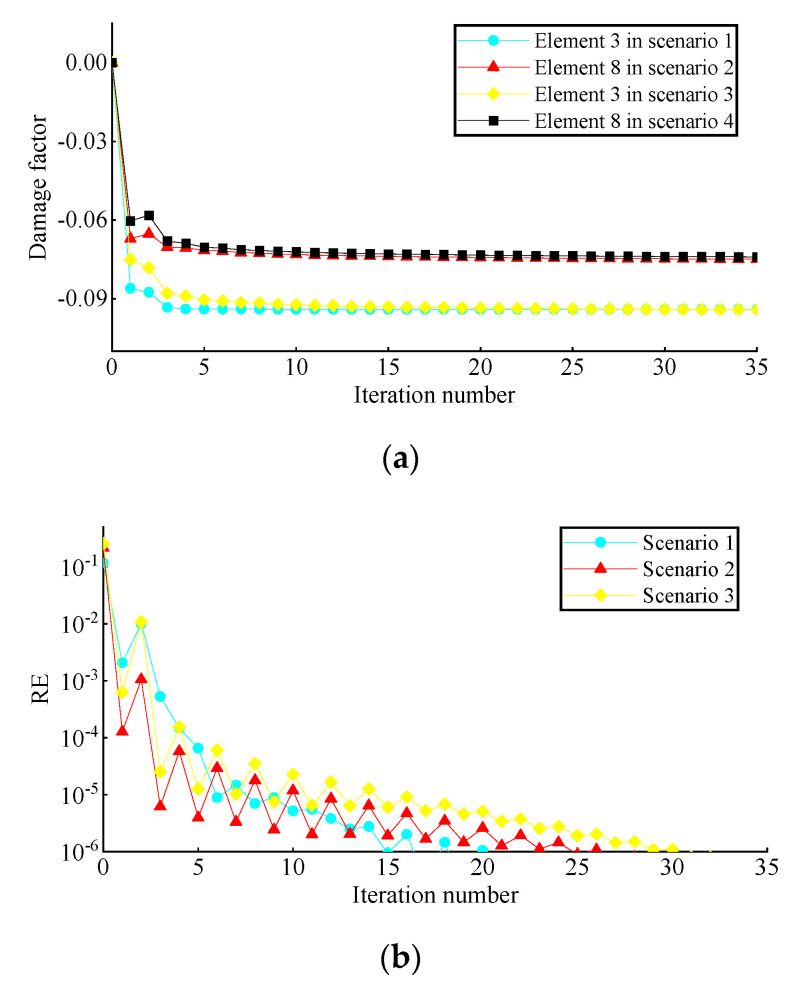
Convergence curve of damage identification: (**a**) damage factor; (**b**) relative error.

**Figure 14:**
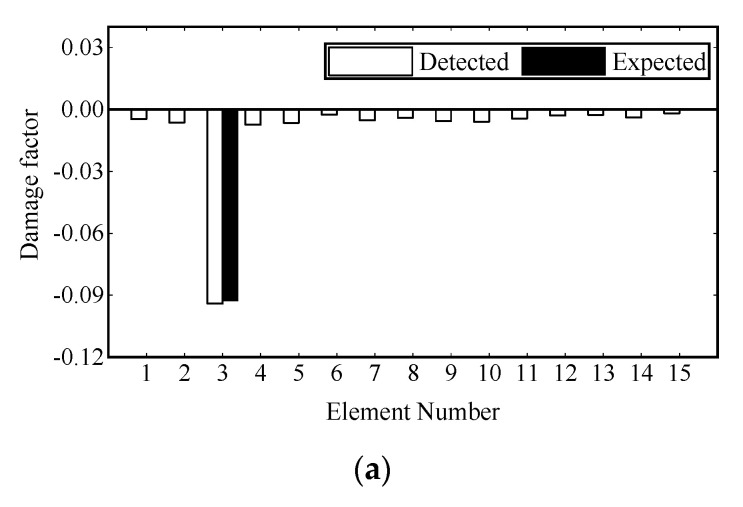

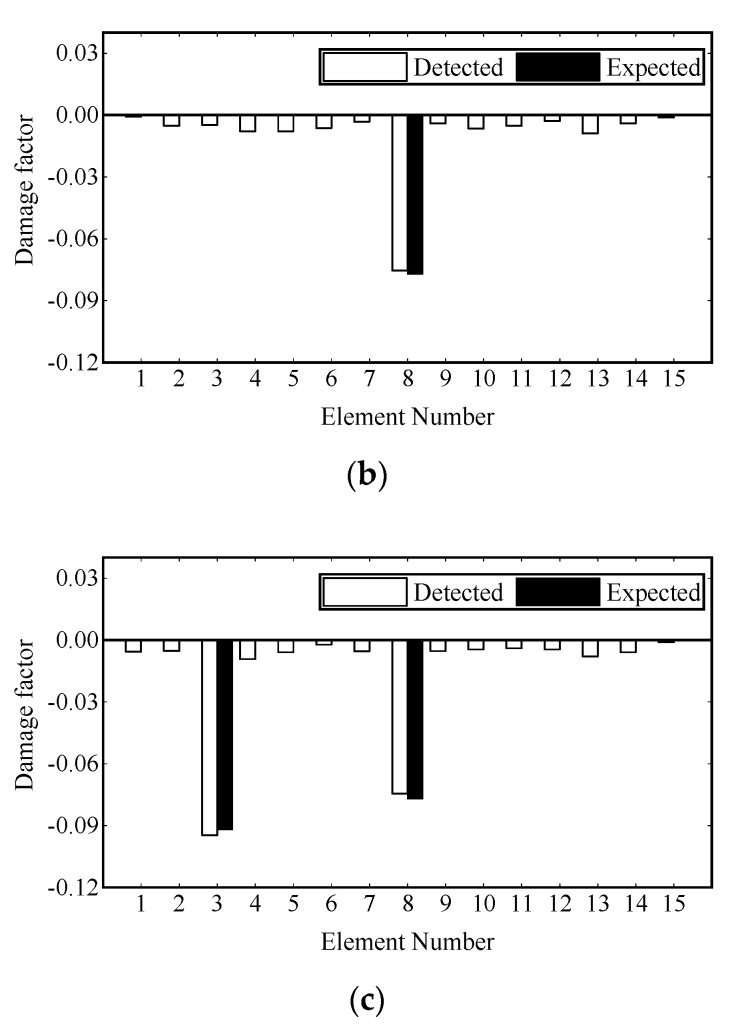
Damage identification results on experimental beams: (**a**) scenario 1; (**b**) scenario 2; (**c**) scenario 3.

Figure 13 shows the convergence curves of the damage factor and RE in the process of FE model updating. Table 4 shows the number of iterations and time consumption for convergence ($\mathrm{RE}\leq 1.0\times 10^{-6}$) under different damage scenarios. It can be seen that the damage factor of the preset damage elements can converge near the expected value in the third iteration, and the fluctuation of RE in the iteration process is mainly caused by the change of damage factors of other elements and gradually tends to decrease gently. The final damage identification results are shown in Figure 14. It is noted that the identification results of the elements with preset damage are in good agreement with the corresponding values given in Table 2, and some minor errors are in the remaining elements. The damage identification results are satisfactory.

## 6. Conclusions

A novel damage identification method based on transmissibility in the time domain is proposed. In the optimization process, combined with the EMD-based dynamic response reconstruction method, the relevant parameters corresponding to the damage state are solved by minimizing the difference between the reconstructed data and the measured data of the sensor position and updating the finite element model. Simulation studies and experimental tests are conducted on a simply supported overhanging beam. The effect of measurement noise is investigated by numerical analysis. The effectiveness and accuracy of the proposed method are demonstrated experimentally.

Based on a theoretical study, numerical simulations and validation experiments, some conclusions can be obtained as follows:

(1) In this study, a novel strategy of damage identification in the time domain is proposed. Compared with the existing damage identification method, the proposed method uses the internal relationship between two locations in the structure as the basis of damage identification. The damage identification can be located and quantified in the time domain without modal identification and frequent time–frequency conversion, which can greatly improve efficiency on the premise of ensuring accuracy. It is suitable to identify the structural damage under transient excitation or stochastic excitation that can excite the modal response of the structure.
(2) According to numerical analysis results, the accuracy of damage identification under different noise levels and excitation types can be guaranteed. Although the recognition accuracy will be affected under a high noise level, it can still accurately locate the damaged element. The proposed method has good noise resistance and robustness.
(3) The experimental beam corresponding to the simulation case verifies the effectiveness and accuracy of the damage identification method. Under the three damage scenarios, the damage factors converge stably and rapidly.

Future works will focus on applying this method to structures in the presence of closely spaced modes. The research on the optimization of sensor layouts is also ongoing.

## Figures and Tables

**Table 1:** Damage scenarios.

Damage Scenario	Damage Description
Scenario 1	Section loss of Element 3
Scenario 2	Section loss of Element 8
Scenario 3	Section loss of Element 3 and Element 8

**Table 2:** Calculation results of equivalent damage.

Calculated Method	Scenario 1	Scenario 2	Scenario 3	
Element number	3	8	3	8
Calculated value	−0.0926	−0.0771	−0.0920	−0.0771

**Table 3:** Specifications of the strain gauge.

Properties	Value
Type	BF350-3AA strain gauge
Resistance (Ω)	349.8 ± 0.1
Sensitivity coefficient	2.1 ± 0.1
Substrate size	7.1 mm × 4.5 mm
Grid size	5.0 mm × 3.0 mm
Grid material	Constantan
Limited strain	2.0%

**Table 4:** The number of iterations and time consumption for convergence under different damage scenarios.

Damage Scenario	Scenario 1	Scenario 2	Scenario 3
Number of iterations	21	27	31
Time consumption (s)	0.476	0.597	0.657

## Data Availability

Not applicable.

## Nomenclature

*n*	number of damage parameters	Abbreviation	
*Nt*	number of time points	FE	finite element
*Nr*	number of transmissibility	RE	relative error
**ε**	strain response vector	DOF	degree of freedom
**Q**	modal coordinate vector	EMD	empirical mode decomposition
$\eta_{i}$	the i-th modal response vector	Superscripts or subscripts	
**K**, **M**	stiffness matrix, mass matrix	T	transpose of matrix or vector
**B**	strain-displacement matrix	*a*, *b*	location index
*J*	objective function	*j*	the *j*-th mode
‖⋅‖	Frobenius norm	(*l*)	the *l*-th element
$\lambda_{s}$, **Λ**	the *s*-th eigenvalue, eigenvalue matrix	*k*	the *k*-th iteration
**W**	weighting matrix	*r*	reconstructed modal response
**S**	sensitivity matrix	*f*	modes of FE model
*α*_l_, **α**	damage factor of l-th element, damage factor vector	m	modal response extracted from measured response
*ψ*, **Ψ**	strain modal contribution, strain modal matrix	*d*	modes and modal response of actual structure
